# Kystique intra-péritonéal et maladie de Crohn: à propos d’une association exceptionnelle

**DOI:** 10.11604/pamj.2018.30.48.14601

**Published:** 2018-05-18

**Authors:** Wafa Ben Ameur, Lamia Kallel, Houcine Maghrebi, Slim Haouet, Azza Filali

**Affiliations:** 1 Service de Gastroentérologie A, Hôpital la Rabta, Tunis, Tunisie; 2 Service de Chirurgie Générale et Digestive A, Hôpital la Rabta, Tunis, Tunisie; 3 Service d’Anatomopathologie, Hôpital la Rabta, Tunis, Tunisie

**Keywords:** Lymphangiome, maladie de Crohn, anti-TNF alpha, Lymphangioma, Crohn's disease, anti-TNF alpha

## Abstract

Le lymphangiome kystique est une tumeur bégnine malformative rare des vaisseaux lymphatiques à localisations diverses. La localisation intra-abdominale du lymphangiome kystique est moins fréquente comparée à la localisation cervico-axillaire. Sa présentation clinique est polymorphe. Le diagnostic est évoqué par l'imagerie mais il nécessite une confirmation histologique. Le traitement de choix est chirurgical. Nous rapportons ici un cas rare de lymphangiome kystique intrapéritonéale acquis suite à une colectomie subtotale chez une patiente porteuse de maladie de Crohn à géni évolutif sévère et ayant nécessité l'introduction des anti TNF alpha. La patiente s'est présentée pour une masse para médiane droite douloureuse irréductible et non impulsive à la toux faisant retenir le diagnostic d'une éventration étranglée sur cicatrice de laparotomie médiane et amenant à opérer la patiente en urgence. L'exploration chirurgicale a montré une masse multi-kystique intra péritonéale extériorisée en partie à travers l'éventration paramédiane droite. L'examen anatomo-pathologique a permis de confirmer le diagnostic de lymphangiome kystique. Devant une exérèse chirurgicale incomplète, la patiente a eu deux séances de ponction évacuatrice en post-opératoire. C'est le premier cas de lymphangiome kystique rapporté chez un patient sous Anti-TNF alpha. Ceci pourrait être expliqué par la perturbation du système immunitaire et plus spécifiquement la population lymphocytaire. Cette association n'a pas été jusque-là prouvée et des études expérimentales sont nécessaires pour affirmer ou infirmer cette hypothèse.

## Abstract (en)

Cystic lymphangioma is a rare benign malformative tumor of the lymphatic vessels which may occur in various locations. Intra-abdominal cystic lymphangioma is less frequent than cervicoaxillary cystic lymphangioma. Clinical presentation is polymorphic. Diagnosis is based on imaging data but it requires histological confirmation. Surgery is the gold standard treatment. We here report a rare case of acquired intraperitoneal cystic lymphangioma secondary to subtotal colectomy in a female patient with severe evolutive Crohn’s disease treated with anti-TNF alpha. The patient presented with irreducible right painful paramedian mass with no impulse on coughing, suggesting the diagnosis of strangulated eventration within a surgical scar from midline laparotomy. She underwent emergency surgery. Surgical exploration showed multi-cystic intraperitoneal mass protrunding through the right paramedian eventration. Anatomo-pathological examination helped to confirm the diagnosis of cystic lymphangioma. Postoperatively, the mass was punctured twice to evacuate the fluid, because of incomplete surgical resection. This is the first reported case of cystic lymphangioma in a patient under anti-TNF alpha. It could be caused by disruption of the immune system and more specifically of the lymphocyte population. This association has not hitherto been established and experimental studies are necessary to accept or refuse this hypothesis.

## Introduction

Le lymphangiome kystique est une affection bénigne dont l'origine serait malformative et qui se révélerait le plus souvent au bas âge et siège au niveau de la région cervico-axillaire. D'autres localisations, en l'occurrence intra-abdominales, mésentériques, rétropéritonéales ou épiploïques sont possibles, mais demeurent moins communes. De nombreuses complications peuvent survenir au cours de l'évolution du lymphangiome kystique, liées à sa localisation et à son volume. Il est donc essentiel de faire le diagnostic de cette masse afin d'éviter la survenue d'une complication abdominale (ischémie mésentérique, volvulus, souffrance intestinale, etc.).

## Patient et observation

Une patiente âgée de 39 ans, thyroïdectomisée en 1999 sous traitement substitutif, est suivie depuis 1992 pour une maladie de Crohn colique compliquée en 2005 d'une colite aigüe grave cortico-résistante pour laquelle elle avaitbénéficié d'une colectomie subtotale. Le rétablissement de la continuité par anastomose iléo-sigmoïdienne a été réalisé en 2009 en raison de phénomènes inflammatoires marqués au niveau du sigmoïde restant, malgré l'introduction de l'imurel depuis 2006. La patiente a développé une énorme éventration sur sa cicatrice de laparotomie. Sur le plan ano-périnéal, la patiente a présentéune fistule ano-vulvaire complexe productive qui a été drainée par séton et traitée par Infliximab puis de l'Humira à partir de 2011, devant une perte de réponse. En Décembre 2011 et sous combothérapie, la patiente a rapporté une extravasation intermittente chronique de liquide séreux mais parfois purulent, à travers la cicatrice de laparotomie, sans accélération du transit ni fièvre, sans syndrome inflammatoire (CRP = 5mg/l, globules blancs à 3800/mm^3^, taux de plaquettes normal) avec un discret syndrome carentiel (anémie ferriprive à 10,4g/dl d'hémoglobine, albumine à 34g/l). Une entéro IRM a été réalisée éliminant une fistule entéro-cutanée et montrant des anses iléales non distendues, à paroi fine, avec une anse anastomotique prenant discrètement le contraste par gadolinium sans épaississement pariétal ni signe inflammatoire en regard, partiellement herniée à travers la cicatrice de l'orifice de stomie au niveau de la fosse iliaque droite. La cicatrice médiane était le siège de quelques trajets d'allure fibreuse en hyposignal T2 sans prise de contraste après injection. Il y avait par ailleurs une petite formation kystique oblongue de 3 cm pariétale droite, entre le muscle grand droit et le tissu cellulo-graisseux, sans collection profonde ni épanchement intra-péritonéal. L'endoscopie basse a conclu à des lésions de recto-sigmoïdite en poussée minime. Une cure de ciprofloxacine de 10 jours a été prescrite et la patiente est restée stable sur le plan luminal et ano-périnéal.

**Figure 1:**
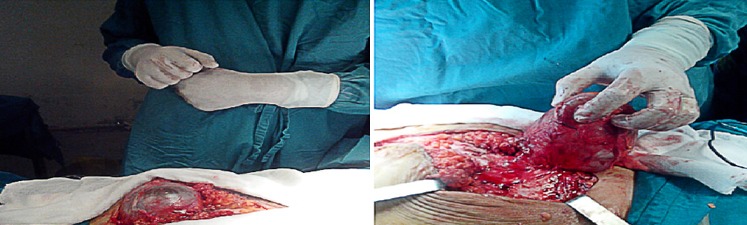
Masse kystique intra péritonéale

**Figure 2:**
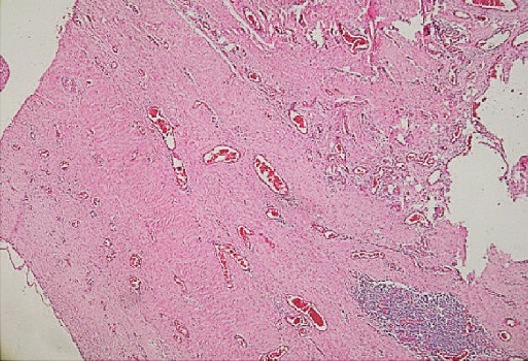
Coupe histologique de la paroi kystique

**Figure 3:**
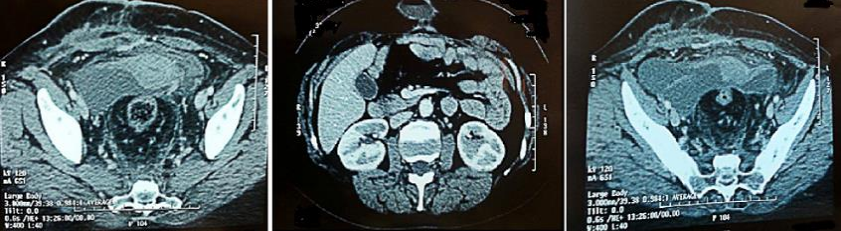
Collection pariétale de 4,5cm à contenu hydroaérique et 2 formations kystiques multi loculées latéro-utérines mesurant 15cm à droite et 10cm à gauche

Elle a été hospitalisée en urgence en Mars 2014, dans un tableau de douleurs abdominales aigues sans arrêt des matières et des gaz. L'examen clinique notait la présence d'une masse para médiane droite douloureusede 15cm de diamètre, irréductible et non impulsive à la toux sans fièvre ni syndrome inflammatoire biologique faisant retenir le diagnostic d'une éventration étranglée sur cicatrice de laparotomie médiane et amenant à opérer la patiente en urgence. La reprise de l'incision médiane, a permis de découvrir une masse kystique extériorisée à travers l'éventration paramédiane droite faisant 10 cm de diamètre à contenu séreux communiquant avec un deuxième kyste intra péritonéal de 3,5cm (Figure 1). Il a été procédé à une ponction aspiration du contenu ramenant un liquide séreux puis résection de la paroi kystique et mise en place d'une plaque pour renforcement pariétal. L'étude anatomopathologique a montré une paroi kystique épaisse inflammatoire, richement vascularisée, contenant de nombreux amas lymphoïdes, quelques macrophages spumeux et des fibres musculaires lisses, sans toutefois voir de revêtement propre sur les coupes disponibles. Il n'avait pas de signes histologiques de malignité avec à l'étude immuno- histochimique,des anticorps CD34 et Calrétinine négatifs, permettant d'éliminer un mésothéliome kystique et un hémangiome caverneux, le tout étant très en faveur d'un lymphangiome kystique (Figure 2). L'exploration a été complétée, deux semaines plus tard, par une TDM abdominale qui a montré une importante infiltration de la paroi abdominale avec présence d'une collection de 4 cm à contenu hydroaérique. Par ailleurs, il existait 2 formations kystiques multi loculées latéro-utérines bilatérales mesurant 15cm à droite et 10cm à gauche pouvant cadrer avec des kystes vestigiaux plutôt qu'avec un hydrosalpinx (Figure 3), sans épanchement intra-péritonéal. Depuis, la patiente a eu deux séances de ponction évacuatrice du kyste pariétal et du kyste latéro-utérin gauche, ramenant à chaque fois un liquide séreux contenant 20g/l d'albumine, acellulaire.

## Discussion

Le lymphangiome kystique est une tumeur bénigne rare, beaucoup plus fréquente chez l'enfant que chez l'adulte. Les localisations habituelles sont cervicales ou axillaires (95% des cas), plus rarement au niveau médiastinal ou abdominal (5-10% des cas). En ce qui concerne la région abdominale, il touche préférentiellement le mésentère et le rétropéritoine, en raison d'une grande richesse du réseau lymphatique [1]. La présentation clinique non spécifique et polymorphe du lymphangiome kystique est liée au volume tumoral, à la localisation et aux types de complications qu'il engendre (mécanique/infectieuse/hémorragique) [2,3]. La physiopathogénie du lymphangiome kystique reste incomplètement élucidée, mais peut toutefois s'expliquer par un défaut congénital ou acquis du drainage lymphatique. La destruction des canaux lymphatique peut être secondaire à des traumatismes physiques tel que la chirurgie ou la radiothérapie [1]. Nous rapportons ici un cas rare de lymphangiome kystique intrapéritonéale acquis suite à une colectomie subtotale chez une patiente porteuse de maladie de crohn à géni évolutif sévère et ayant nécessité l'introduction des anti TNF alpha. Quelques cas de lymphangiomes kystiques intra abdominaux ont été rapportés dans la littérature dont un cas de lymphangiome épiploïque acquis suite à une résection partielle du grand épiploon dans le cadre d'une gastrectomie partielle pour un cancer de l'estomac. Cette résection partielle du grand épiploon pourrait expliquer le développement du lymphangiome chez notre patiente dans les suites de la colectomie subtotale sachant que l'omentectomie est fortement recommandée en cas de colectomie. C'est le premier cas de lymphangiome kystique rapporté chez un patient sous biothérapie (Anti-TNF alpha). Cette dernière, par la perturbation du système immunitaire et plus spécifiquement la population lymphocytaire qu'elle engendre, aurait-elle un rôle favorisant dans le développement de cette tumeur. Cette association n'a pas été jusque-là prouvée et des études expérimentales sont nécessaires pour affirmer ou infirmer cette hypothèse.

Le diagnostic du lymphangiome kystique n'est pas toujours évident, mais l'échographie est l'examen utile initialement et pour le suivi, elle montre une masse creusée de cavités kystiques à contenu liquidien hypoéchogène, de tailles variables et à parois fines (hémodynamiquement inactive au doppler). Le scanner est un excellent moyen diagnostique complémentaire. Il montre une tumeur homogène, hypo dense, à cloisons fines, non rehaussée par le contraste [4,5]. L'IRM est utilisée uniquement en seconde intention, elle permet une étude plus précise des rapports anatomiques de la lésion avec les structures avoisinantes. On peut également avoir recours à des techniques plus invasives telles qu'une ponction à l'aiguille fine du liquide intra-kystique et l'examen cytologique de ce dernier révélant la présence de lymphocytes. Néanmoins, la certitude diagnostique est apportée par l'analyse anatomopathologique de la tumeur. A l'histologie, trois critères sont nécessaires au diagnostic: 1) il s'agit d'une formation kystique; 2) les cloisons sont constituées d'un stroma conjonctif pourvu de tissu lymphoïde et de muscle lisse; 3) le kyste est bordé d'un revêtement endothélial à type lymphatique (positivité du facteur D2-40) démontrant l'origine vasculaire de la tumeur [6-9]. L'étude en immunohistochimie est positive pour le facteur CD 31 et l'actine [10]. En cas de découverte fortuite, l'abstention thérapeutique avec un suivi régulier est conseillée si le lymphangiome kystique est asymptomatique. Une régression spontanée peut se voir dans 1,6 à 16% des cas. L'exérèse chirurgicale est l'attitude classique, car il existe un risque élevé d'évolution de la lésion et de complications. La résection chirurgicale doit être totale et la plus conservatrice. Il existe un taux de récidive de 40% après résection incomplète et de 17% après exérèse macroscopiquement complète [3]. L'aspiration du contenu du kyste avec ou sans injection de produit sclérosant reste une alternative thérapeutique pour les tumeurs non résécables.

## Conclusion

Le lymphangiome kystique est une tumeur bénigne rare évolutive nécessitant une exérèse chirurgicale totale afin d'éviter au maximum toute progression et récidive. La confirmation diagnostique est apportée uniquement par l'analyse anatomopathologique de la tumeur.

## Conflits d’intérêts

Les auteurs ne déclarent aucun conflit d'intérêts.
